# Hematuria and Kidney Failure in a Patient with Kidney Cancer

**DOI:** 10.34067/KID.0000000674

**Published:** 2025-05-29

**Authors:** Yoko Fujita, Shiika Watanabe, Daisuke Ichikawa

**Affiliations:** 1 Division of Nephrology and Hypertension, Department of Internal Medicine, St. Marianna University School of Medicine, Kawasaki, Japan; 2 Department of Internal Medicine, Yokohama General Hospital, Yokohama, Japan

**Keywords:** AKI, kidney cancer, thrombosis

## Abstract

**Figure:**
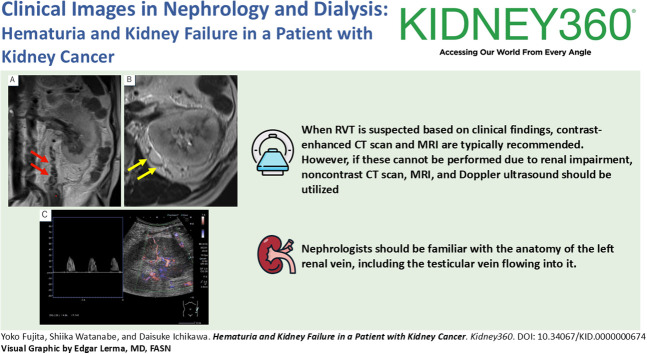

## Case Description

A 71-year-old man with a history of diabetes mellitus and hypertension had previously undergone a total right nephrectomy and partial left nephrectomy for renal cell carcinoma (clear cell carcinoma) 6 months before admission. Two months before his current visit, he was diagnosed with pyelonephritis and worsening kidney function, as indicated by increased perirenal fat concentrations on noncontrast computed tomography (CT) scan and a significant inflammatory response in laboratory tests. Antibiotic treatment was initiated, but a urine culture was negative. Serum creatinine levels continued to rise, and hematuria developed, and noncontrast magnetic resonance imaging (MRI) image showed no signs of cancer recurrence. Suspecting rapidly progressive GN, the patient was referred to our hospital.

On admission, the patient's serum creatinine had increased to 6.08 mg/dl (baseline: 1.5 mg/dl). Urinalysis revealed nonglomerular hematuria and proteinuria. P-ANCA, C-ANCA, antinuclear antibodies, and complement levels were all within normal ranges. Kidney biopsy was not performed due to the prior nephrectomy. D-dimer level was elevated at 4.8 *μ*g/ml.

**Figure 1:**
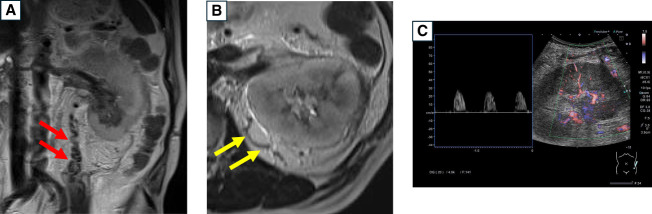
**The findings of noncontrast MRI and Doppler ultrasound which suggest renal vein thrombosis.** (A and B) magnetic resonance imaging showing enlargement of the (A) left testicular vein and (B) renal capsular vein. (C) Doppler ultrasound of the interlobar artery showing systolic phase-only blood flow.

Noncontrast MRI showed enlargement of the left testicular vein and the renal capsular vein (Figure 1, A and B). Noncontrast CT scan revealed an enlarged left kidney, a dilated left renal vein with high density, and increased perirenal fat concentrations. Doppler ultrasound of the interlobar artery indicated systolic phase-only blood flow, suggesting the presence of renal congestion due to thrombosis (Figure 1C). Renal vein ultrasonography showed an embolus on the aortic side of the left testicular vein branch.

Based on these findings, the patient was diagnosed with AKI due to renal vein thrombosis (RVT). After the initiation of antithrombotic therapy, serum creatinine level decreased to 2.68 mg/dl. However, 8 months later, a recurrence of left renal cancer was detected, with the thrombosed area showing progression, raising the possibility of malignancy-related thrombosis.

## Discussion

This report describes a case of a 71-year-old man with a history of renal cell carcinoma who developed rapid kidney dysfunction, likely caused by RVT. Noncontrast CT scan, MRI, and Doppler ultrasound were crucial for the diagnosis.^1,2^

RVT is a rare condition that can lead to renal dysfunction and may present with microscopic hematuria and severe proteinuria. Common causes of RVT include nephrotic syndrome, hypercoagulability, malignancy, compression of the renal veins, trauma, and postrenal transplantation.^2^ Active malignancy is reported to be the most common cause of RVT^3^ and should always be considered in the differential diagnosis.

Contrast-enhanced CT scan is typically the preferred imaging modality for diagnosing RVT due to its high sensitivity and specificity.^2^ However, contrast-enhanced CT scan may not always be feasible in cases of renal dysfunction. In this patient, MRI revealed marked dilatation of the left testicular vein alongside renal enlargement, suggesting RVT. When hematuria and renal enlargement are observed in a patient with unexplained renal failure, RVT should be considered, and further investigation is warranted.

## Teaching Points

- When RVT is suspected based on clinical findings, contrast-enhanced CT scan and MRI are typically recommended. However, if these cannot be performed due to renal impairment, noncontrast CT scan, MRI, and Doppler ultrasound should be used.
- Nephrologists should be familiar with the anatomy of the left renal vein, including the testicular vein flowing into it.
